# Hepatic DNA damage in harbour porpoises (*Phocoena phocoena*) stranded along the English and Welsh coastlines

**DOI:** 10.1002/em.22205

**Published:** 2018-07-03

**Authors:** Karina Acevedo‐Whitehouse, Kathy J. Cole, David H. Phillips, Paul D. Jepson, Rob Deaville, Volker M. Arlt

**Affiliations:** ^1^ Institute of Zoology, Zoological Society of London London United Kingdom; ^2^ Unit for Basic and Applied Microbiology Autonomous University of Querétaro Querétaro Mexico; ^3^ Section of Molecular Carcinogenesis Institute of Cancer Research Sutton Surrey United Kingdom; ^4^ Department of Analytical, Environmental and Forensic Sciences, MRC‐PHE Centre for Environment and Health King's College London London United Kingdom; ^5^ NIHR Health Protection Research Unit in Health Impact of Environmental Hazards at King's College London in partnership with Public Health England London and Imperial College London United Kingdom

**Keywords:** DNA damage, harbour porpoise, genotoxicity, persistent organic pollutants (POPs), polychlorinated biphenyls (PCBs), polycyclic aromatic hydrocarbons (PAHs), ^32^P‐postlabeling

## Abstract

One level at which persistent organic pollutants (POPs) and polycyclic aromatic hydrocarbons PAHs) can exert damage is by causing DNA strand‐breaks or nucleotide base modifications, which, if unrepaired, can lead to embryonic mutations, abnormal development and cancer. In marine ecosystems, genotoxicity is expected to be particularly strong in long‐lived apex predators due to pollutant bioaccumulation. We conducted ^32^P‐postlabeling analyses optimized for the detection and quantification of aromatic/hydrophobic DNA adducts in the livers of 40 sexually‐mature North Atlantic harbour porpoises (*Phocoena phocoena*) stranded along the English and Welsh coastlines. We examined hepatic tissue to search for inflammatory and preneoplastic lesions and examine their association with adduct levels. Adducts were found in all porpoises (mean: 17.56 ± 11.95 per 10^8^ nucleotides), and were higher than levels reported for marine vertebrates from polluted sites. The pollutants causing the induced DNA adducts could not be further characterized. Hepatic DNA damage did not correlate with levels of blubber POP concentrations (including total polychlorinated biphenyl [PCBs], dichlorodiphenyltrichloroethane [DDT] and dieldrin); PAH concentrations were not available for the present study. However, DNA damage predicted occurrence of inflammatory and preneoplastic lesions. Further, our data showed a reduction in hepatic DNA adduct levels with age in the 40 animals examined while POP concentrations, particularly PCBs, increased with age. Using a different dataset of 145 mature male harbour porpoises confirmed that higher contaminant levels (total PCBs, DDT and dieldrin) are found in older animals. The reduction in hepatic DNA adduct levels in older animals was in accordance with other studies which show that suppression of hepatic CYP1A enzyme activity at high PCB concentrations might impact on CYP1A‐mediated DNA adduct formation of PAHs which are ubiquitous environmental pollutants and readily metabolized by CYP1A to species binding to DNA. In summary, our study shows that pollutant‐induced DNA damage is prevalent in harbour porpoises from UK waters and may lead to detectable sub‐lethal hepatic damage. Environ. Mol. Mutagen. 59:613–624, 2018. © 2018 The Authors Environmental and Molecular Mutagenesis published by Wiley Periodicals, Inc. on behalf of Environmental Mutagen Society

## INTRODUCTION

The sustained input of persistent organic pollutants (POPs) into the oceans raises concern about population‐level genotoxic effects in marine wildlife, particularly in productive coastal waters [Ashauer et al. 2006]. Levels of POPs magnify as they move up in the food chain, bioaccumulating in the adipose tissue, and have been linked to diverse systemic disorders including damage to the nervous system, reproductive impairment and disruption to the immune system [Heiden et al. 2008]. More recently, experimental studies have demonstrated genotoxic effects of POPs, evidenced as single or double strand DNA breakage or covalent binding to nucleotides to form chemically‐stable modified bases (‘adducts’). If unrepaired, such damage can lead to gamete loss, lethal embryonic mutations, abnormal development, cancer, heritable mutations or changes in gene expression. Within the marine ecosystem, POP genotoxicity is likely to pose a serious threat to the survival and persistence of long‐lived top predators [Depledge 1998], such as marine mammals. Some of the highest recorded levels of POPs have been recorded in marine mammals over the past several decades [Braune et al. 2005; Blasius and Goodmanlowe 2008]. Owing to their apex position in the trophic chain [Aguilar and Borrell 2005], lipid‐rich blubber that acts as a reservoir for lipophilic chemicals, and limitations in their metabolic and excretory capacity [Ruus et al. 2002], marine mammals are at a high risk of POP bioaccumulation and, by inference, its harmful effects.

To date, the majority of studies on POP‐genotoxicity in marine organisms have centred on invertebrates and, to a lesser extent, on teleost fish, whereas effects in marine mammals have yet to be studied in depth. This taxonomic bias has been due, at least in part, to experimental exposure trials in marine mammals being precluded and to the difficulty of obtaining fresh tissues for analysis. A notable exception to the paucity of similar studies in marine mammals is that conducted in beluga whales (*Delphinapterus leucas*) from the St. Lawrence Estuary in Canada. Elevated cancer rates [Martineau et al. 1988] and high levels of industrial pollutants recorded in this population prompted research on causal links between POPs and systemic damage to the beluga whales’ health. Aromatic DNA adducts have been found in liver and brain tissue of dead St. Lawrence belugas [Martineau et al. 1994; Ray et al. 1991], providing evidence of exposure to genotoxic pollutants. Further analyses indicated that DNA adducts were also detectable in belugas from the less‐polluted Canadian Arctic [Mathieu et al. 1997]. Taken together, these observational studies emphasize the need to investigate biological impacts of lethal and sub‐lethal POP‐genotoxicity in marine mammals.

The aim of our study was to assess evidence for genotoxicity induced by environmental exposure to POPs in the harbour porpoise (*Phocoena phocoena*). This cetacean is the most abundant in Northern Europe and is distributed all around the UK coast, where strandings are frequent [Jepson 2005]. Increased interactions with fisheries, habitat degradation, changes in prey availability, pollution and infectious disease have been suggested to have caused harbour porpoise populations to decline in the English Channel and southern North Sea. The species’ wide‐ranging diet puts it at high risk from POP bioaccumulation and deleterious effects. Relatively high concentrations of polychlorinated biphenyls (PCBs) [Jepson et al. 1999; Pierce et al. 2008], fluorinated contaminants such as perfluorooctane sulphonate and perfluorooctanoic acid [Law et al. 2008], organochlorine pesticides [Bruhn et al. 1999; Chu et al. 2003] and traces of unmetabolised polycyclic aromatic hydrocarbons (PAHs) [Law and Whinnett 1992] have been detected in North Atlantic harbour porpoises. High levels of contaminants have been linked to thyroid fibrosis [Schnitzler et al. 2008], higher nematode burden [Bull et al. 2006] and mortality due to infectious disease [Jepson et al. 1999; Jepson et al. 2005] in harbour porpoises. However, genotoxic effects induced by constant environmental exposure to marine xenobiotics still remain unexplored.

In order to investigate the prevalence of hepatic POP‐induced genotoxicity in sexually mature harbour porpoises, we conducted ^32^P‐postlabeling analyses optimized to quantify bulky aromatic/hydrophobic DNA adducts. We also examined hepatic tissue for changes (e.g. parenchymal hyperplasia, portal fibrosis and bile duct proliferation) that have been associated with organic pollutants in marine fish [Myers et al. 1994; Akcha et al. 2003] and carnivores [Reichert et al. 1998; Sonne et al. 2008]. We tested the predictions that (*i*) females have less POP‐related DNA damage (measured as bulky/hydrophobic hepatic adducts) than males, owing to a partial transfer of their contaminant load to their offspring during lactation [Ruus et al. 2002], (*ii*) DNA damage is higher in older individuals, as would be expected if DNA repair mechanisms of porpoises become less efficient with age [Gorbunova et al. 2007], and (*iii*) the frequency of hepatic lesions is higher in individuals with higher DNA damage.

## MATERIAL AND METHODS

### Study Design

**Figure 1:**
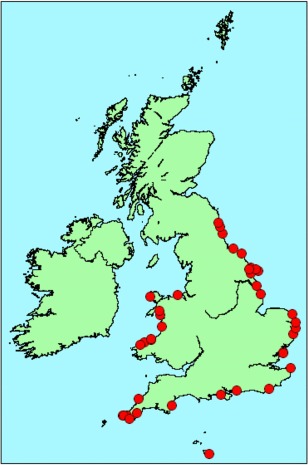
Stranding locations of the common 40 harbour porpoises (*Phocoena phocoena*) included in this study.

The study was carried out in co‐operation with the UK Cetacean Strandings Investigation Programme (UK CSIP), which has been responsible for investigating causes of morbidity and mortality in UK stranded cetaceans since 1990. For our analyses, we selected 40 sexually mature harbour porpoises (17 female and 23 male), which had been found stranded along the English and Welsh coastlines, between 1991 and 2001. Figure 1 shows the sampling collection. The 40 individuals included in the study were selected rigorously based on the freshness of their post‐mortem condition (Condition code 2; [Jepson 2005]), to control for the effects of lipid mobilization due to autolysis. Only individuals that were considered extremely fresh (Condition code 2a “as if just died, no bloating, meat is considered by most to be edible”) or showing slight decomposition (Condition code 2b “slight bloating, blood imbibition visible”) were selected for analyses. For all porpoises, liver samples had been collected during necropsy and frozen at –80°C immediately following collection. All porpoises had been weighed, measured, sexed by anatomical examination, and aged by quantification of growth‐layer groups from decalcified tooth sections [Lockyer 1995]. A variety of POPs, including PCBs, dichlorodiphenyltrichloroethane (DDT) dichloro‐2,2‐bis(p‐chlorophenyl)ethylene (DDE) and dieldrin, had been measured in their blubber as part of other toxicology studies and the raw data were available for our study. Briefly, blubber concentrations of 25 individual chlorinated biphenyl (∑25CB) congeners were measured on a wet‐weight basis with protocols routinely used in the United Kingdom government Centre for Environment, Fisheries and Aquaculture Science (CEFAS) laboratory. A certified reference material (European Community Bureau of Reference 349 [cod liver oil]) was analysed alongside the blubber samples (see detailed months in [Jepson et al. 2005]). The sum of the concentrations of the 25 CB congeners determined (∑25CB) were then converted to a lipid basis (mg/kg lipid) using the proportion of hexane extractable lipid (%HEL) in individual blubber samples. The individual International Union of Pure and Applied Chemistry (IUPAC) CB congeners analysed were numbers 18, 28, 31, 44, 47, 49, 52, 66, 101, 105, 110, 118, 128, 138, 141, 149, 151, 153, 156, 158, 170, 180, 183, 187, and 194. Dieldrin, DDT and DEE (as mg/kg lipid) were measured as described [Jepson 2005]. For each porpoise, the thickness (in mm) of the ventral blubber layer was measured [Jepson et al. 2005].

### Assessing DNA Damage: ^32^P‐Postlabeling

DNA was extracted from 40 archived liver samples that had been collected during necropsy and immediately frozen at −80°C for long‐term storage (see above). Each sample, consisting of approximately 300 mg of liver, was thawed and macerated in a sterile microcentrifuge tube and then incubated for 6 hours at 60°C with proteinase K‐SDS‐Tris HCl buffer (pH 8.0). Genomic DNA was isolated by a standard phenol‐chloroform extraction method [Green and Sambrook 2012]. DNA concentration was determined by spectrophotometry. In all cases, the A_260_/A_280_ ratio ranged between 1.7 and 1.9.

DNA adducts were measured for each sample using the nuclease P_1_ enrichment version of the ^32^P‐postlabeling method as described previously [Tang et al., 2001; Arlt et al. 2008]. Briefly, 4 μg of DNA per sample were digested with micrococcal nuclease (120 mU, Sigma‐Aldrich, UK) and calf spleen phosphodiesterase (40 mU, Calbiochem, UK), enriched and labelled with [γ‐^32^P]ATP as reported. Polyethyleneimine‐cellulose (Macherey‐Nagel, Düren, Germany) thin‐layer chromatography (TLC) was used to resolve adducts using the following conditions: D1, 1.0 M sodium phosphate, pH 6; D3, 3.5 M lithium‐formate, 8.5 M urea, pH 3.5; D4, 0.8 M lithium chloride, 0.5 M Tris, 8.5 M urea, pH 8. After chromatography, TLC plates were scanned using a Packard Instant Imager (Downers Grove, IL, USA) and DNA adduct levels (RAL, relative adduct labelling) were calculated from the adduct cpm, the specific activity of [γ‐^32^P]ATP, and the amount of DNA (pmol of DNA‐P) used. All samples were analysed in triplicate on different days. An external benzo[*a*]pyrene‐7,8‐dihydrodiol‐9,10‐epoxide‐(BPDE)‐modified DNA standard was used as a positive control. As in prior studies, total DNA adduct levels were measured in the diagonal radioactive zone (DRZ) area of the TLC plates and were considered representative of PAH‐DNA and other aromatic/hydrophobic adducts resistant to nuclease P_1_ digestion [Tang et al. 2001; Kim et al. 2011; Long et al. 2016]. The method provides a summary measure of a complex mixture of adducts present in the postlabeling chromatograms. The detection limit of the ^32^P‐postlabeling assay was ∼1 adduct per 10^10^ normal nucleotides. Results were expressed as total adducts/10^8^ nucleotides.

### Hepatic Lesions

We analysed the necropsy reports for evidence of macroscopic hepatic lesions and conducted microscopic examination of the archived liver sections of the 40 porpoises here studied. The tissues had been collected at necropsy and immediately fixed in 10% formalin. Fixed tissue was embedded in paraffin, sliced at 3 μm of thickness, and stained with haematoxylin‐eosin. Liver sections were examined by microscopy for the presence of liver flukes (*Campula oblonga*) lesions and unspecific changes, including parenchymal hyperplasia, bile duct proliferation and portal fibrosis [Reichert et al. 1998; Sonne et al. 2008]. Two slides were examined per each individual. We did not find evidence of hepatic neoplasm in any of the samples.

### Statistical Analyses

To correct for deviations from normality and heteroscedasticity, all response variables were examined using Shapiro‐Wilk normality tests and square‐root or log transformed when necessary. A linear regression model was used to examine whether age and sex could explain a significant proportion of the variance in DNA damage. A generalized linear model was used to test whether DNA adducts predicted hepatic lesions. Occurrence of lesions was expressed as a binary response (0 = no evidence of infection, 1 = evidence of infection). This model was constructed using a binomial error structure and a logit link. Significance testing was carried out using F‐tests to compensate for overdispersion [Crawley 2002]. All tests were conducted within the program R version 2.5.0 (http://www.r-project.org) [Ihaka and Gentleman 1996].

## RESULTS

### Hepatic DNA Adducts

**Figure 2:**
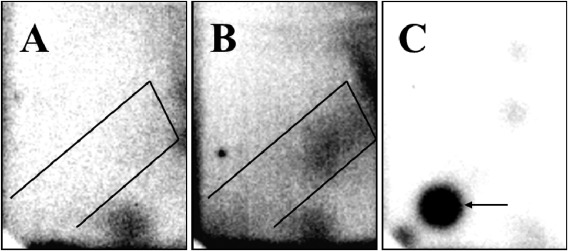
Representative autoradiographs of thin‐layer chromatograms of ^32^P‐postlabeling digests of liver DNA from harbour porpoises: (A) liver sample with low DNA damage; (B) liver sample with high DNA damage. The marked area shows the diffuse diagonal radioactive zone (DRZ) in liver DNA used for quantitation. (C) Positive control using a benzo[*a*]pyrene‐7,8‐dihydrodiol‐9,10‐epoxide (BPDE)‐modified DNA standard; the arrow shows the 10‐(deoxyguanosin‐*N* ^2^‐yl)‐7,8,9‐trihydroxy‐7,8,9,10‐tetrahydrobenzo[*a*]pyrene (dG‐*N* ^2^‐BPDE) adduct.

Bulky aromatic and/or hydrophobic DNA adducts were found in all liver samples (Table 1). This was manifested in the appearance of weak diagonal radioactive zones (DRZs) on TLC plates on which the labelled DNA digests had been subjected to multidirectional chromatography (Fig. 2A and 2B). The typical area of the DRZ on the chromatogram used for the quantitation of DNA adduct levels is marked on the autoradiogram (see Fig. 2A and 2B). DNA adduct levels ranged from 3.5 to 55.6 adducts/10^8^ nucleotides (mean = 17.57, SD = 11.95). DNA binding data showed a positively‐skewed distribution (Shapiro‐Wilk normality test; W = 0.8896, *p*‐value < 0.001), and were log‐transformed before conducting further analyses. As levels of DNA adducts did not vary between stranding year (ANOVA; F_5,39_ = 0.58, *P* = 0.71) nor stranding location (ANOVA; F_3,39_ = 0.36, *P* = 0.77), samples were considered as a single group for all further statistical analyses.

**Table 1:** Levels of DNA adducts in harbour porpoise livers and other study parameters (age, sex, pollutant exposure and histopathology)

Ref. Code	Age of animal	Sex of animal	DNA adduct levels/10^8^nucleotides (mean ± SD) in diagonal radioactive zone (DRZ)^a^	∑25 CBcongeners (mg/kg lipid)in blubber	DDT(mg/kg lipid)in blubber	Dieldrin (mh/kg lipid)in blubber	Hepatic hyperplasia not associated wth Liver fluke (*Campula oblonga*)infection
SW1999/172	8	Male	3.5 ± 1.1	17.72	0.75	1.29	0
SW1994/7A	5	Female	4.2 ± 2.1	15.16	0.55	1.04	0
SW1991/14	14	Female	5.8 ± 0.3	138.75	0.39	8.75	0
SW1990/94	11	Female	6.2 ± 4.4	32.53	5.33	8.80	0
SW1997/1	6	Male	6.3 ± 0.8	29.45	1.26	3.22	0
SW1992/198	15	Male	6.6 ± 2.2	58.21	3.13	8.37	0
SW1991/17	8	Male	6.7 ± 2.3	46.18	0.13	7.08	0
SW1997/118	8	Female	6.9 ± 3.1	5.29	0.24	0.27	NA
SW1999/10	5	Female	7.6 ± 3.3	1.52	0.07	0.12	0
SW1991/19A	5	Male	7.7 ± 3.0	35.22	0.13	6.22	0
SW2001/149	8	Female	7.7 ± 3.1	12.94	0.44	1.09	0
SW1993/31	18	Male	7.9 ± 3.1	37.39	2.74	5.59	0
SW1996/160	14	Female	8.1 ± 2.2	2.35	0.16	0.17	0
SW1994/44	4	Male	8.2 ± 1.7	45.74	4.55	13.33	0
SW2000/146(2)	5	Male	8.3 ± 0.7	16.53	0.52	1.97	0
SW1996/46	7	Male	9.7 ± 2.7	46.35	2.19	4.39	1
SW1994/7	10	Male	12.1 ± 3.7	39.66	NA^b^	NA	0
SW1994/185	5	Female	13.2 ± 2.2	18.21	2.38	3.69	1
SW1991/36	14	Female	13.6 ± 4.2	12.90	0.12	1.94	0
SW1991/104	12	Male	13.8 ± 2.0	67.56	NA	NA	NA
SW1995/61	7	Female	14.2 ± 2.6	21.40	NA	NA	0
SW1996/37	9	Male	15.7 ± 0.1	82.33	NA	NA	0
SW1991/116	5	Female	16.8 ± 7.2	32.56	NA	NA	NA
SW1995/86	5	Female	17.5 ± 3.2	7.64	NA	NA	1
SW1994/171	2	Male	18.5 ± 7.4	45.62	3.61	16.86	0
SW1996/163	6	Male	19.2 ± 4.8	54.65	1.19	4.13	0
SW1991/22	11	Male	21.5 ± 2.3	22.35	0.04	0.19	1
SW1993/12	8	Male	22.2 ± 7.3	17.87	NA	NA	0
SW1991/24	7	Male	23.1 ± 5.0	41.54	0.15	6.59	1
SW1997/36	7	Female	24.6 ± 3.5	11.84	0.46	1.15	0
SW1994/53	3	Female	25.9 ± 2.9	2.47	NA	NA	0
SW1992/9	14	Male	26.8 ± 10.8	95.59	NA	NA	1
SW1993/27	6	Female	29.6 ± 1.8	78.29	NA	NA	NA
SW1992/165	6	Male	31.2 ± 13.5	150.47	NA	NA	1
SW1993/20	5	Male	31.7 ± 14.3	38.69	NA	NA	0
SW1996/2	5	Male	33.8 ± 4.5	7.46	NA	NA	1
SW1998/149	4	Female	36.2 ± 2.6	14.97	0.80	0.96	0
SW1995/84	5	Female	36.6 ± 0.7	3.12	NA	NA	0
SW1996/27(1)	5	Male	37.9 ± 6.7	43.33	NA	NA	1
SW2000/150A	4	Male	55.6 ± 1.4	43.58	0.66	3.21	0

a Each DNA sample was analysed by three independent ^32^P‐postlabeling assays.

b Not analysed.

We did not find a significant relationship between blubber POP concentrations (Σ25CB congeners, DDT and dieldrin) and levels of hepatic DNA damage in the analysed samples (for all independent linear regression models, *P* > 0.1).

### The Effect of Sex and Age on Hepatic DNA Damage

**Figure 3:**
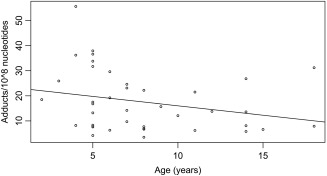
Levels of hepatic DNA damage according to the age of harbour porpoises. As DNA adduct levels did not conform to expectations of normality and homoscedasticity, statistical analyses were conducted using log‐transformed DNA adduct data (adducts/10^8^ nucleotides) Age explained 10% of the variance in DNA adduct levels recorded for 40 individuals (adjusted *R* ^2^: 0.098; *P* = 0.029). Sex did not contribute significantly in the model.

Contrary to our predictions, DNA adduct levels (log transformed number of adducts/10^8^ nucleotides) did not vary between sexes (*P* > 0.1), and although age was a significant predictor of DNA damage, explaining 10% of the variance in adduct levels (see full model in Table 2), these tended to decrease with age rather than increase (Fig. 3). This may indicate less efficient hepatic detoxification of pollutants in older animals, instead of reduction in their DNA repair capacity. Under this scenario, individuals who become less efficient at metabolizing genotoxic hydrophobic pollutants would predictably maintain higher levels of these contaminants in their tissues, while forming less DNA adducts due to decreased enzymatic transformation.

**Table 2:** Full linear model of hepatic DNA damage (measured as mean DNA adduct levels/10^8^ nucleotides) in harbour porpoises

Explanatory variable^a^	Estimate	*F*	d.f.	*P*
Age	−0.03955	4.9564	1	0.032
Sex (factor)	−0.07708	0.4797	1	0.493
Age:Sex	0.01872	0.5399	1	0.467

a Explanatory variables included in the model are age and sex. When non‐significant terms were removed from the model, age explained 10% of the variance in DNA adduct levels (*P* = 0.029).

**Figure 4:**
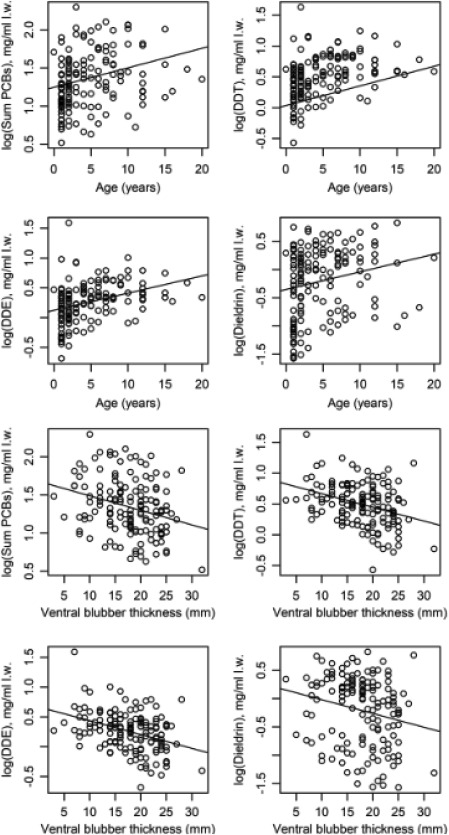
Relationship of age (top half of figure) and ventral blubber depth (bottom half of figure) with PCB, DDT, DDE and dieldrin concentrations in adult male harbour porpoises; *n* = 145 (data source: UK CSIP database). Pollutant data are shown as log‐transformed values (mg/kg lipid weight). Values for age (years) and blubber thickness (mm) correspond to the same individuals Please see Table III for statistical analysis of charts. Data on contaminant levels for the 145 adult male harbour porpoises is presented in Supporting Information Table I.

To partially address this *a posteriori* hypothesis, we analysed data that was available from previous studies for 145 sexually mature male harbour porpoises. For each animal there were data on blubber concentration of total PCBs, DDT, DDE and dieldrin, age and blubber depth (data source: UK CSIP database; see Supporting Information Table I). The data had been collected in the exact same way as described above (see methods) for the 40 animals included in our study. Independent linear regression models were then used to examine whether age (years) and/or ventral blubber depth (mm) could explain a significant proportion of the variance in contaminant concentrations. Contaminant concentrations were log‐transformed prior to running the analyses. As expected, both age and blubber‐depth were independent predictors of contaminant levels; older animals and thinner animals both revealed higher (more concentrated) contaminant levels (Fig. 4; see full models in Table 3). According to the UK CSIP database the maximum life span in UK‐stranded harbour porpoises is around 20–22 years.

**Table 3:** Full linear models for contaminant concentrations in adult male harbour porpoises

Term^a^	Estimate	*F*	df	*P*	Adjusted *R* ^2^
PCB					
Age	0.2373	15.44	1	0.0001	0.09
Blubber	−0.6231	10.69	1	0.0013	0.06
DDT					
Age	0.3867	38.85	1	<0.0001	0.16
Blubber	−0.6914	21.43	1	<0.0001	0.12
DDE					
Age	0.3419	32.26	1	<0.0001	0.19
Blubber	−0.7286	25.28	1	<0.0001	0.09
Dieldrin					
Age	0.4648	13.97	1	0.0002	0.08
Blubber	−0.7009	5.48	1	0.0205	0.02

a Terms included in the model are age and sex. All dependent variables are log‐transformed. Blubber = blubber depth. Age:blubber interactions were non‐significant in all models and were removed from final calculations. Age explained 4–14% of the variance in contaminant levels, while blubber depth explained 4–14% of the variance in contaminant levels (see Fig. 2). Data on contaminant levels for the 145 adult male harbour porpoises is presented in Supporting Information Table I.

### DNA Damage and Hepatic Lesions

**Figure 5:**
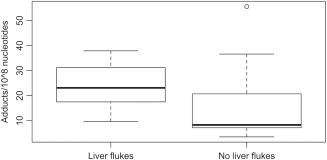
Average levels of liver DNA damage measured as bulky aromatic adducts in harbour porpoises that had hepatic lesions that were unrelated to liver fluke infection (*n* = 8) and in those that had hepatic lesions that were clearly associated with liver fluke infection (*n* = 28). The difference is statistically significant (*P* < 0.02) between both groups.

Thirty‐nine of the 40 harbour porpoises (98%) examined had chronic pericholangitis with varying degrees of periportal fibrosis, bile duct hyperplasia and lymphocytic infiltration, all of which can be caused by liver fluke (*Campula oblonga*) infections [Siebert et al. 2006]. The one harbour porpoise that did not show any evidence of hepatic pathology had the lowest level of DNA damage (3.5 adducts/10^8^ nucleotides). Because *C. oblonga* infection and their commonly associated proliferative lesions were so widespread in the harbour porpoises included in our study, it was difficult to determine in which cases these lesions could be unequivocally classified as independent of fluke infection. To circumvent this problem we grouped porpoises that presented hepatic abnormalities (bile duct proliferation and portal fibrosis as well as non‐inflammatory changes) but no evidence of liver flukes and no lymphocytic infiltration, and classified them as having lesions that were plausibly non‐infectious in origin. DNA damage was a significant predictor of such lesions (GLM, F_2,39_ = 5.80, *P* = 0.02); affected individuals (*n* = 8) showing an increased number of DNA adducts than the rest of the porpoises (Fig. 5). Age and sex were not significant explanatory factors and were removed from the final model (data not shown).

## DISCUSSION

The aim of our study was to assess the occurrence of DNA damage related to environmental exposure of harbour porpoise to POPs and other environmental pollutants (e.g. PAHs). Using ^32^P‐postlabeling analyses that were optimized for the detection of bulky hydrophobic/aromatic adducts, we quantified damage in the liver samples of 40 porpoises. The levels of hepatic DNA adducts reported here are generally lower than those reported for two species of mullet in analyses carried out in this laboratory [Telli Karakoc et al. 1997]. Results are broadly similar to those for beluga whales from heavily‐polluted regions [Martineau et al. 1988; Ray et al. 1991; Mathieu et al. 1997], and for a number of teleost fish species from polluted sites around the English and Welsh coastlines (e.g. [Lyons et al. 2000; 2006]) and the Swedish Baltic coast (e.g. [Sundberg et al. 2007]). Comparisons between studies should be treated with caution, however, especially as they may involve different marine species and varying ^32^P‐postlabeling conditions methods may have been used in each laboratory.

Adducts in isolated DNA are fairly stable at long‐term storage, although it has been discussed that confounding I‐compounds (Indigenous covalent DNA modifications) might be formed in degraded or improperly preserved tissues and interfere with the interpretation of ^32^P‐postlabeling analyses [Reichert et al. 1999]. Based on meticulous revision of UK CSIP necropsy reports, we ensured that all the samples included in our study had been collected from fresh carcasses, that is those considered to be Condition 2 according to the criteria used to categorize cetacean carcass condition for post‐mortem analyses (extremely fresh or slight decomposition; [Jepson 2005]), and that the collected liver section had been frozen at −80°C immediately after collection and maintained at this temperature until the analyses were conducted. Thus, we are confident that there was minimal or no interference from artefacts and that the DNA adducts detected are true measures of DNA damage induced by hydrophobic/aromatic compounds. Chemical pollutants, including those with genotoxic effects, continue to be introduced into the coastal marine environment by industrial and urban discharge, agricultural run‐off and large‐scale industrial accidents. A large number of aromatic pollutants such as PAHs are mutagenic and carcinogenic and undergo metabolic activation before they can react with DNA and exert genotoxicity [Arlt et al. 2008; Wohak et al., 2016]. Furthermore, various PCB congeners (*e.g*. mono‐ di‐, tri‐, and tertrachlorobiphenyls) are capable of forming DNA adducts [Ludewig and Robertson 2013; IARC 2016]. Thus there is evidence that for certain PCBs both covalent DNA adduct formation and generation of reactive oxygen species must be considered equally plausible modes of action. PCB‐induced DNA adducts are detectable by TLC ^32^P‐postlabeling [Oakley et al. 1996; McLean et al. 1996; Schilderman et al., 1999, 2000; Pereg et al. 2002; Borlak et al. 2003]. Importantly previous studies have shown that DNA adducts induced by PCBs such as 2‐chloro‐, 3,4‐dichloro, 2,4,4′‐trichloro‐, 3,4,5‐trichloro‐ and 2,2′,5,5′‐tetrachlorobiphenyl are detectable under the chromatography solvent conditions used for ^32^P‐postlabeling in the present study [Schilderman et al. 2000]. As shown in Figure 2 this leads to a diffuse zone of radioactivity in a diagonal band on the chromatograms (called the DRZ, diagonal radioactive zone) which is indicative for the presence of bulky DNA adducts derived from aromatic and hydrophobic compounds. While the ^32^P‐postlabeling method is optimized to detect DNA adducts caused by aromatic and hydrophobic compounds [Phillips and Arlt 2007], it does not identify which specific compound(s) was responsible for their accumulation in the hepatic cells. Hydrophobic DNA adducts reflect a substantial range of responses to the exposure to complex mixtures of contaminants including those derived from PCBs and other polyhalogenated aromatic hydrocarbons. However, it is difficult to separate PCB‐induced DNA adducts from PAH‐derived DNA adducts [Schilderman et al. 2000]. Nevertheless, studies have shown for example that total DNA adduct levels can serve as a dosimeter for internal dose of co‐occurring PAHs and PCBs in hepatopancreatic tissue of crayfish collected in the river Meuse [Schilderman et al. 1999].

Previous studies on contaminants in harbour porpoises from UK waters have reported that high tissue concentrations of aromatic and hydrophobic contaminants such as PCBs and PAHs are common [Jepson et al. 1999; Law et al. 2006; Law et al. 2008], thus implying that exposure to those contaminants is widespread. According to the UK CSIP which assessed PCBs in cetaceans (primarily harbour porpoises) from 1990 to 2008 mean concentrations by area were: Scotland 11.5 mg/kg lipid weight; East, 16.0 mg/kg lipid weight; and West, 20.5 mg/kg lipid weight [Law et al., 2012]. We did not observe a significant association between DNA adducts and blubber contaminant (PCB, dieldrin and DDT) concentrations in the animals included in this study. Thus, PCB concentrations may represent a general measure of chronic exposure to a complex mixture of environmental pollutants but it is reasonable to postulate that the DNA adducts observed in the harbour porpoises’ hepatic cells may be caused by exposure to PAHs and/or other aromatic compounds as yet unmeasured in the harbour porpoise tissues. Data on PAHs in harbour porpoises from UK waters are scarce. One study found that total PAH ranged from 0.11‐0.56 µg/g wet weight of muscle tissue of harbour porpoises; 2–4 ring PAHs such as naphthalenes, phenanthrenes, anthracene, fluoranthene and pyrene were detectable but no higher PAHs like benzpyrenes [Law and Whinnett 1992]. In this respect, the use of other techniques such as immunochemical detection of PAH‐specific DNA damage in target tissues [Machella et al. 2005] or measurement of CYP1A1 expression [Wilson et al. 2005], as well as controlled laboratory exposures of porpoise hepatic cells to individual contaminants or mixtures of the specific components, might help to identify the exact compound(s) responsible for the adducts observed. However, it still cannot be excluded that PCBs contribute to the observed hepatic DNA damage in the present study.

The levels of hepatic DNA adducts result from a balance between their formation through metabolic activation of aromatic and hydrophobic compounds, and their loss through DNA‐repair mechanisms, which include rate of repair and expression of genes encoding for DNA repair enzymes or DNA damage response elements, and through apoptosis. Thus, DNA damage might be expected to be more evident in older animals if DNA repair processes became less efficient with increasing age, a phenomenon that has been observed in experimental studies on mice [Gorbunova et al. 2007]. In addition, as marine predators are constantly exposed to POPs, and as the tissue concentrations of POPs increase with age [Whyte et al. 2000], higher levels of DNA damage might be predicted due to cumulative exposure, particularly in males, as they cannot decrease their contaminant burden during lactation as females do [Ross et al. 2000; Tanabe et al. 1988]. A study of California sea lions (*Zalophus californianus*) provided some support for the ‘senescence of DNA repair processes’ hypothesis by showing that male DNA adduct levels increased proportionally to total body length (as an indirect estimate of age) [Reichert et al. 1999]. However, our results showed the opposite pattern; with DNA adduct levels being significantly higher in younger porpoises. Therefore, it is possible that the lower levels of DNA damage observed in older individuals reflect decreased metabolic capacity rather than impairment of DNA repair processes.

Age‐related reduction in hepatic metabolism has been observed in vertebrates, including fish and mice, where older animals show reduced activities of hepatic biotransformation enzymes, such as ethoxyresorufin‐O‐deethylase (EROD; a measure for cytochrome P450 [CYP] 1A enzyme activity), and reduced xenobiotic metabolism, presumably from down‐regulation of gene expression [Couillard et al. 2004; Kashiwada et al. 2007]. Some studies have indicated the possible involvement of PCB congeners in the inhibition of CYP1A enzyme activity (EROD) in flounder (*Phatichthys flesus*) or teleost scup (*Stenotomus chrysops*) [Besselink et al., 1998; Schlezinger and Stegeman, 2001]. Similar findings were also reported in Atlantic tomcod (*Microgadus tomcod*) at high PCB concentrations [Couillard et al., 2005]; Tomcod were studied at three locations along the Canadian east coast and for the three sites combined CYP1A enzyme activity EROD) decreased as PCB levels increased. It could be that our results are evidence of a similar phenomenon. Thus, it is tempting to speculate that individuals who are less efficient at metabolizing genotoxic pollutants will maintain higher levels of these contaminants in their tissues while forming less DNA adducts. To address this possibility, we analysed the UK CSIP database to examine the association between age and levels of various POPs in adult male harbour porpoises, finding a significant positive linear relationship with age. This result adds strength to our suggestion that detoxification of pollutants decreases with age and subsequently more un‐metabolized compounds can be accumulated. Evidently, with the available data, it is difficult to account for bioaccumulation of xenobiotics due to continued exposure [Depledge 1998; Aguilar and Borrell 2005], which would also increase concentrations in older animals. Future analyses of hepatic enzyme activities and gene expression in harbour porpoises or other top‐predator cetaceans will be fundamental to understanding the age‐related differences in DNA adduct levels that we found in our study.

Unrepaired DNA damage caused by genotoxic pollutants can lead to mutation and cancer [Myers et al. 1994; Akcha et al. 2003]. We hypothesized that hepatic lesions would be more frequent in harbour porpoises with higher levels of DNA damage. Regrettably, the high prevalence of liver fluke infections, which cause proliferative hepatic pathology [Siebert et al. 2006], made it difficult to determine unambiguously whether these lesions could have been induced by DNA damage. Additive or synergistic effects of chronic inflammation caused by liver fluke infection combined with genotoxicity could potentially enhance cancer risk in the porpoise liver. However, it is interesting to note that those porpoises with proliferative lesions whose necropsy reports did not indicate liver fluke infection and which had no lymphocytic infiltration had significantly higher levels of hepatic DNA damage than the porpoises whose lesions were clearly associated with flukes. Furthermore, the single harbour porpoise that did not have any hepatic pathology had the lowest levels of DNA damage. It is possible that the high levels of unrepaired DNA adducts are leading to hepatic damage in the harbour porpoise, as has been observed in other species [Myers et al. 1994: Akcha et al. 2003]. A larger data set would be needed to test this hypothesis more rigorously.

## CONCLUSIONS

Our study provides evidence that pollutant‐induced DNA damage is widespread among harbour porpoises from UK waters. Average levels of hydrophobic/aromatic DNA adducts were comparable to what has been reported for other vertebrate species from heavily polluted areas. Adduct levels were higher in younger porpoises, suggesting that the capacity to metabolize hydrophobic/aromatic compounds might decrease with age. Furthermore, porpoises with hepatic proliferative lesions but no evidence of liver fluke infections had higher levels of DNA damage than those animals whose lesions were associated with liver flukes, demonstrating associations between pollutant‐induced DNA adduct formation and hepatic tissue lesions. Our study highlights the need for further research to improve our understanding of the prevalence of genotoxic damage and a wider range of biological effects caused by chronic exposure to environmental pollutants such as POPs and PAHs. ^32^P‐postlabeling analysis applied to health studies of marine top predators may prove to be an extremely sensitive and logistically‐feasible method for detecting and monitoring the sub lethal effects of environmental genotoxic pollutants.

## AUTHOR CONTRIBUTIONS

KAW, PDJ and RD provided the tissue samples, conducted the histopathology and POP analyses. KJC, DHP and VMA conducted the DNA adduct analysis. KAW and VMA initiated the study, had the main responsibility for the study design, interpretation and for finalizing the manuscript. DHP was involved in the study design, interpretation and contributed to the writing of the manuscript. All authors critically reviewed the manuscript and approved it.

## Supporting information

Supporting Information
