# Supplementary material for: Hepatic DNA damage in harbour porpoises (Phocoena phocoena) stranded along the English and Welsh coastlines
Source: Environ Mol Mutagen. 2018 Jul 3;59(7):613–24. doi: 10.1002/em.22205 (PMC6174976; doi:10.1002/em.22205)
Supplement: Supplementary file 1 — Supporting Information [file EM-59-613-s001.docx]

**SUPPLEMENTARY TABLE I**. **Age and blubber levels of persistent organic contaminants in 145 male harbour porpoises. Data from UK Cetacean Strandings Investigation Programme database, and previously published by Jepson et al. 2005 and Law et al. 2012.**

| **Ref. Code** | **Age (years)** | **Ventral blubber depth (mm)** | **∑25 CB congeners (mg/kg lipid) in blubber** | **DDT (mg/kg lipid) in blubber** | **DDE (mg/kg lipid) in blubber** | **Dieldrin (mg/kg lipid) in blubber** |
| --- | --- | --- | --- | --- | --- | --- |
| SW1992/157A | 8 | 14 | 12.81 | 3.76 | 2.24 | 0.13 |
| SW1992/168A | 5 | 12 | 23.31 | 7.42 | 4.16 | 0.27 |
| SW1992/208A | 4 | 20 | 22.79 | 6.49 | 2.87 | 0.31 |
| SW1993/64D | 12 | 21 | 15.44 | 3.91 | 2.09 | 0.30 |
| SW1994/165B | 7 | 21 | 20.5 | 4.32 | 2.56 | 0.28 |
| SW1995/120B | 7 | 14 | 7.70 | 1.89 | 1.20 | 1.52 |
| SW1995/131A | 16 | 18 | 15.60 | 3.43 | 1.87 | 0.15 |
| SW1995/141 | 1 | 16 | 18.89 | 2.97 | 1.70 | 1.70 |
| SW1996/101 | 2 | 3 | 30.50 | 3.65 | 1.86 | 2.21 |
| SW1996/119 | 2 | 20 | 39.88 | 8.89 | 4.51 | 1.59 |
| SW1996/147A | 12 | 18 | 13.05 | 3.55 | 1.98 | 1.40 |
| SW1996/163 | 6 | 15 | 109.12 | 6.85 | 3.26 | 4.13 |
| SW1996/169A | 6 | 19 | 5.86 | 1.33 | 0.84 | 0.66 |
| SW1996/174 | 2 | 21 | 37.53 | 2.55 | 1.52 | 0.84 |
| SW1996/44 | 1 | 20 | 26.23 | 5.08 | 3.00 | 2.78 |
| SW1996/50B | 4 | 18 | 4.60 | 1.02 | 0.57 | 0.52 |
| SW1996/60 | 8 | 18 | 98.23 | 4.12 | 2.13 | 2.13 |
| SW1996/60B | 1 | 19 | 5.37 | 1.30 | 0.76 | 0.07 |
| SW1996/67 | 1 | 23 | 16.99 | 4.34 | 2.93 | 2.61 |
| SW1996/84B | 1 | 20 | 5.80 | 1.09 | 0.66 | 0.09 |
| SW1996/84E | 2 | 16 | 6.45 | 1.47 | 0.98 | 0.68 |
| SW1996/86B | 9 | 14 | 21.32 | 6.42 | 3.70 | 3.48 |
| SW1996/87 | 1 | 9 | 69.35 | 13.19 | 9.57 | 5.65 |
| SW1997/1 | 6 | 21 | 55.76 | 8.62 | 5.86 | 3.22 |
| SW1997/124A | 8 | 14 | 14.23 | 3.31 | 2.09 | 1.63 |
| SW1997/135F | 2 | 18 | 9.70 | 3.07 | 2.11 | 1.33 |
| SW1997/141 | 2 | 19 | 11.02 | 2.27 | 1.33 | 1.57 |
| SW1997/142 | 4 | 17 | 39.76 | 6.33 | 3.33 | 2.78 |
| SW1997/161A | 12 | 20 | 10.43 | 2.15 | 1.40 | 0.14 |
| SW1997/173 | 2 | 15 | 55.50 | 4.67 | 2.42 | 1.87 |
| SW1997/174B | 1 | 18 | 11.81 | 2.41 | 1.44 | 0.19 |
| SW1997/178C | 4 | 14 | 35.08 | 6.59 | 3.75 | 2.73 |
| SW1997/186(1) | 1 | 18 | 11.98 | 2.27 | 1.43 | 1.21 |
| SW1997/2 | 2 | 20 | 23.43 | 1.60 | 0.92 | 0.62 |
| SW1997/67F | 1 | 9 | 16.96 | 4.44 | 3.08 | 1.67 |
| SW1997/87 | 2 | 18 | 19.93 | 4.02 | 2.33 | 1.33 |
| SW1997/93B | 3 | 25 | 5.84 | 1.35 | 0.86 | 0.53 |
| SW1997/97A | 11 | 23 | 5.29 | 1.28 | 0.88 | 0.75 |
| SW1998/1 | 1 | 25 | 19.94 | 1.69 | 0.99 | 0.62 |
| SW1998/115 | 15 | 19 | 101.87 | 10.80 | 5.57 | 6.70 |
| SW1998/123A | 15 | 18 | 13.01 | 3.99 | 2.61 | 1.30 |
| SW1998/149 | 4 | 18 | 29.55 | 3.12 | 1.74 | 0.97 |
| SW1998/164 | 5 | 12 | 69.17 | 6.75 | 2.80 | 4.19 |
| SW1998/170 | 4 | 8 | 81.23 | 4.90 | 3.15 | 1.74 |
| SW1998/174 | 7 | 17 | 127.45 | 11.8 | 6.18 | 4.49 |
| SW1998/183 | 2 | 16 | 33.45 | 3.41 | 1.20 | 1.41 |
| SW1998/18B | 8 | 19 | 35.58 | 7.75 | 4.27 | 3.03 |
| SW1998/208 | 2 | 22 | 8.16 | 1.86 | 1.21 | 0.90 |
| SW1998/21 | 1 | 20 | 17.62 | 0.79 | 0.45 | 0.45 |
| SW1998/56A | 18 | 10 | 30.42 | 6.06 | 3.84 | 0.21 |
| SW1998/58A | 5 | 21 | 4.27 | 1.05 | 0.65 | 0.71 |
| SW1998/71 | 2 | 18 | 17.68 | 3.24 | 1.87 | 1.62 |
| SW1998/76 | 6 | 24 | 9.76 | 2.16 | 1.32 | 0.88 |
| SW1999/121B | 4 | 24 | 10.02 | 1.65 | 1.01 | 1.25 |
| SW1999/148A | 9 | 16 | 27.83 | 6.85 | 3.48 | 2.02 |
| SW1999/172 | 8 | 16 | 34.61 | 3.44 | 2.04 | 1.29 |
| SW1999/180A | 12 | 28 | 66.16 | 14.57 | 6.20 | 5.87 |
| SW1999/25A | 5 | 24 | 9.67 | 2.43 | 1.59 | 0.09 |
| SW1999/48C | 12 | 10 | 15.73 | 3.78 | 2.44 | 1.86 |
| SW1999/57A | 5 | 20 | 7.16 | 1.45 | 0.88 | 0.99 |
| SW1999/60 | 1 | 25 | 10.35 | 0.67 | 0.46 | 0.34 |
| SW1999/63 | 2 | 24 | 12.03 | 0.52 | 0.33 | 0.33 |
| SW1999/77 | 2 | 27 | 15.04 | 1.76 | 1.10 | 0.81 |
| SW1999/8B | 12 | 23 | 62.14 | 6.03 | 2.64 | 3.08 |
| SW1999/96C | 20 | 20 | 22.45 | 3.89 | 2.18 | 1.61 |
| SW2000/103 | 8 | 20 | 25.85 | 2.80 | 1.28 | 1.40 |
| SW2000/140 | 3 | 16 | 25.89 | 5.42 | 2.92 | 2.58 |
| SW2000/144 | 5 | 18 | 14.56 | 3.23 | 2.05 | 1.82 |
| SW2000/145A | 7 | 9 | 40.46 | 6.55 | 4.57 | 1.91 |
| SW2000/146(1) | 1 | 24 | 19.70 | 2.28 | 1.58 | 1.05 |
| SW2000/146(2) | 5 | 20 | 31.50 | 3.73 | 2.21 | 1.98 |
| SW2000/14A | 10 | 23 | 7.38 | 1.45 | 0.81 | 0.95 |
| SW2000/150A | 4 | 20 | 86.20 | 6.02 | 2.50 | 3.21 |
| SW2000/157 | 1 | 23 | 25.94 | 2.24 | 1.36 | 1.48 |
| SW2000/16 | 3 | 19 | 10.37 | 1.00 | 0.60 | 0.41 |
| SW2000/166 | 5 | 16 | 42.94 | 3.39 | 1.57 | 2.25 |
| SW2000/168 | 4 | 18 | 14.29 | 2.70 | 1.69 | 1.02 |
| SW2000/176 | 2 | 14 | 18.52 | 1.61 | 0.80 | 0.90 |
| SW2000/188A | 1 | 24 | 7.67 | 1.49 | 0.89 | 0.78 |
| SW2000/196 | 7 | 8 | 16.26 | 2.50 | 1.48 | 1.14 |
| SW2000/20 | 3 | 10 | 197.93 | 14.37 | 8.28 | 4.71 |
| SW2000/202A | 3 | 16 | 87.24 | 13.75 | 8.52 | 5.23 |
| SW2000/27 | 1 | 7 | 41.97 | 3.93 | 1.89 | 2.33 |
| SW2000/52A | 2 | 25 | 5.48 | 0.87 | 0.57 | 0.57 |
| SW2000/74A | 2 | 12 | 41.48 | 4.45 | 0.95 | 3.02 |
| SW2000/81A | 2 | 8 | 9.75 | 1.76 | 1.02 | 1.14 |
| SW2001/127 | 4 | 18 | 26.05 | 4.56 | 2.61 | 2.16 |
| SW2001/139 | 10 | 15 | 27.99 | 4.62 | 2.61 | 2.50 |
| SW2001/16A | 7 | 26 | 18.01 | 3.31 | 2.22 | 1.22 |
| SW2001/172A | 12 | 12 | 26.19 | 5.94 | 3.78 | 2.67 |
| SW2001/186 | 5 | 11 | 97.22 | 3.84 | 2.09 | 2.91 |
| SW2001/193 | 10 | 12 | 115.04 | 3.11 | 1.87 | 1.36 |
| SW2001/203 | 3 | 16 | 35.18 | 2.72 | 1.41 | 1.85 |
| SW2001/210 | 1 | 24 | 18.93 | 2.00 | 1.11 | 0.06 |
| SW2001/23 | 10 | 22 | 103.73 | 9.13 | 6.20 | 4.57 |
| SW2001/24A | 3 | 14 | 106.10 | 2.26 | 1.22 | 1.09 |
| SW2001/260 | 3 | 14 | 17.67 | 2.46 | 1.67 | 1.33 |
| SW2001/269A | 7 | 23 | 37.67 | 11.49 | 2.13 | 2.02 |
| SW2001/4 | 2 | 23 | 19.46 | 0.65 | 0.50 | 0.31 |
| SW2001/83A | 1 | 32 | 3.30 | 0.59 | 0.39 | 0.05 |
| SW2001/83D | 6 | 12 | 28.87 | 6.65 | 3.86 | 2.29 |
| SW2001/85D | 0 | 19 | 50.90 | 4.21 | 2.93 | 1.96 |
| SW2001/87A | 3 | 9 | 15.20 | 2.13 | 1.69 | 0.96 |
| SW2001/94 | 1 | 21 | 14.54 | 1.00 | 0.48 | 0.41 |
| SW2001/97A | 1 | 11 | 19.24 | 3.86 | 2.56 | 2.21 |
| SW2002/103 | 1 | 21 | 79.45 | 3.45 | 2.30 | 1.72 |
| SW2002/114 | 1 | 24 | 9.46 | 2.29 | 1.20 | 0.53 |
| SW2002/149B | 1 | 13 | 6.54 | 1.71 | 1.10 | 0.13 |
| SW2002/169A | 12 | 24 | 19.96 | 4.96 | 3.11 | 0.22 |
| SW2002/170 | 2 | 23 | 39.63 | 3.73 | 2.05 | 0.12 |
| SW2002/19 | 7 | 15 | 60.38 | 6.19 | 4.25 | 1.62 |
| SW2002/229 | 2 | 15 | 26.50 | 2.11 | 1.22 | 1.22 |
| SW2002/309 | 6 | 20 | 18.68 | 4.73 | 2.64 | 0.12 |
| SW2002/372A | 7 | 16 | 16.17 | 3.15 | 2.16 | 0.10 |
| SW2002/372C | 1 | 10 | 8.50 | 2.40 | 1.70 | 0.10 |
| SW2002/382 | 2 | 14 | 28.24 | 4.15 | 2.82 | 0.22 |
| SW2003/159A | 8 | 15 | 48.46 | 6.51 | 2.07 | 0.16 |
| SW2003/186 | 15 | 25 | 35.10 | 3.84 | 2.17 | 0.10 |
| SW2003/220 | 2 | 8 | 10.58 | 2.71 | 1.76 | 0.16 |
| SW2003/236 | 2 | 5 | 16.28 | 3.73 | 2.53 | 0.23 |
| SW2003/257C | 1 | 23 | 11.19 | 2.77 | 1.44 | 0.09 |
| SW2003/259 | 2 | 17 | 17.59 | 1.43 | 0.79 | 0.05 |
| SW2003/260 | 3 | 21 | 27.53 | 2.47 | 1.70 | 0.08 |
| SW2003/283 | 2 | 14 | 28.15 | 1.40 | 0.78 | 0.07 |
| SW2003/334 | 10 | 7 | 65.00 | 8.24 | 4.71 | 0.54 |
| SW2003/355C | 1 | 20 | 4.96 | 0.27 | 0.21 | 0.03 |
| SW2003/361 | 1 | 21 | 10.76 | 0.70 | 0.44 | 0.04 |
| SW2003/385 | 10 | 14 | 116.72 | 17.61 | 10.11 | 1.59 |
| SW2003/51A | 9 | 23 | 24.98 | 7.22 | 4.78 | 0.16 |
| SW2004/141A | 1 | 15 | 6.64 | 1.60 | 1.17 | 0.05 |
| SW2004/145 | 2 | 7 | 41.57 | 42.78 | 39.02 | 0.18 |
| SW2004/146 | 1 | 10 | 25.45 | 3.38 | 2.13 | 0.08 |
| SW2004/165 | 1 | 22 | 9.72 | 0.59 | 0.36 | 0.03 |
| SW2004/173C | 6 | 25 | 22.81 | 3.65 | 2.38 | 0.08 |
| SW2004/291C | 6 | 8 | 55.06 | 8.7 | 4.29 | 0.51 |
| SW2004/32A | 8 | 18 | 63.24 | 7.03 | 4.18 | 0.21 |
| SW2004/339 | 3 | 22 | 27.19 | 1.61 | 1.18 | 0.04 |
| SW2004/342 | 1 | 12 | 34.50 | 4.98 | 2.33 | 0.08 |
| SW2004/80 | 1 | 25 | 12.28 | 1.11 | 0.53 | 0.03 |
| SW2004/87 | 1 | 15 | 11.44 | 3.03 | 2.09 | 0.11 |
| SW2004/92A | 2 | 17 | 13.16 | 1.69 | 0.91 | 0.03 |
| SW2005/117A | 1 | 22 | 14.05 | 2.86 | 2.17 | 0.12 |
| SW2005/125A | 1 | 18 | 12.41 | 1.36 | 0.98 | 0.05 |
| SW2005/163A | 7 | 22 | 52.52 | 6.84 | 1.87 | 0.19 |
| SW2005/19D | 6 | 25 | 58.58 | 7.45 | 2.25 | 0.17 |
